# Seizure from water intoxication following bowel preparation: a case report

**DOI:** 10.1186/s12882-022-03035-8

**Published:** 2022-12-15

**Authors:** Ting-Hsuan Chiang, Jui-Hsiang Tan, Chun-Chao Chang, Kuan-Chieh Fang

**Affiliations:** 1 School of Medicine, College of Medicine, Taipei Medical University, Taipei, Taiwan; 2 Division of Gastroenterology, Department of Internal Medicine, Taipei Medical University Hospital, Taipei, Taiwan; 3 Division of Gastroenterology and Hepatology, Department of Internal Medicine, School of Medicine, College of Medicine, Taipei Medical University, Taipei, Taiwan

**Keywords:** Hyponatremia, Colonoscopy, Desmopressin, Bowel preparation, Water intoxication, Case report

## Abstract

**Background:**

Bowel preparation prior to colonoscopic examination is generally considered a safe process. Hyponatremia is a complication that has been reported in literature during bowel preparation. Individuals who develop severe symptomatic hyponatremia are often older and have comorbidities such as hypothyroidism, chronic kidney disease, or adrenal insufficiency. However, other mechanisms and circumstances can also lead to this potentially fatal complication.

**Case presentation:**

We present a unique case of a patient who developed seizure prior to colonoscopy due to acute hyponatremia without any well-known risk factors. With the subsequent diagnosis of water intoxication, the use of desmopressin was believed to have contributed to this serious complication.

**Conclusion:**

In addition to the use of certain well-documented medications and the presence of comorbidities that can lead to hyponatremia, clinicians should also be aware of the use of desmopressin as an important risk factor. Thorough history taking can guide individualized bowel preparation regimens to minimize the risk of undesired complications.

## Background

Bowel preparation with commonly used oral polyethylene glycol (PEG) agent prior to colonoscopy is considered safe and effective by international guidelines [1, 2]. The associated risk of developing electrolyte imbalances such as hyponatremia has been reported up to 6% [3], however, symptomatic hyponatremia is rare and has mainly been documented by case reports. Patient’s age and comorbidities are often believed to directly contribute to the majority of these rare yet severe cases of hyponatremia [4–6]. Although the role of antidiuretic hormone (ADH) in hyponatremia has also been emphasized in existing literature, the use of desmopressin has not been reported to contribute to severe hyponatremia in patients undergoing bowel preparation. Here, we report a unique case of water intoxication after PEG colon preparation that led to severe hyponatremia and subsequently seizure in a patient taking desmopressin.

## Case presentation

A 72-year-old man was scheduled for colonoscopy due to a colonic lesion (lateral spreading tumor at ascending colon) incidentally discovered during a routine health check two months prior. A split-dose PEG lavage was used as the bowel preparation regimen. The night before the colonoscopy, oral prescription of polyethylene glycol (GI klean powder®) was given for bowel preparation. He was advised to increase water intake for adequate bowel prepare and prevention of dehydration. On the day of the colonoscopy, the patient was alert, oriented and denied any discomfort during the morning visit. However, he was found to be lethargic and disoriented a couple hours later during anesthesia evaluation prior to the procedure. In the presence of the anesthesiologist, the patient suddenly developed tonic-clonic seizure. His convulsion spontaneously subsided after approximately one minute. After rapid intubation, he was sent to the intensive care unit for further evaluation.

Upon reviewing the patient’s medical history, he had benign prostate hyperplasia with significant symptoms of urinary frequency and nocturia. For symptom control, he had been taking 0.1 mg of desmopressin and 5 mg of solifenacin once daily for a year. He had a normal serum sodium at 138 mEq/L three weeks before presentation, had no history of seizures, and no family history significant of any specific diseases.

**Fig. 1:**
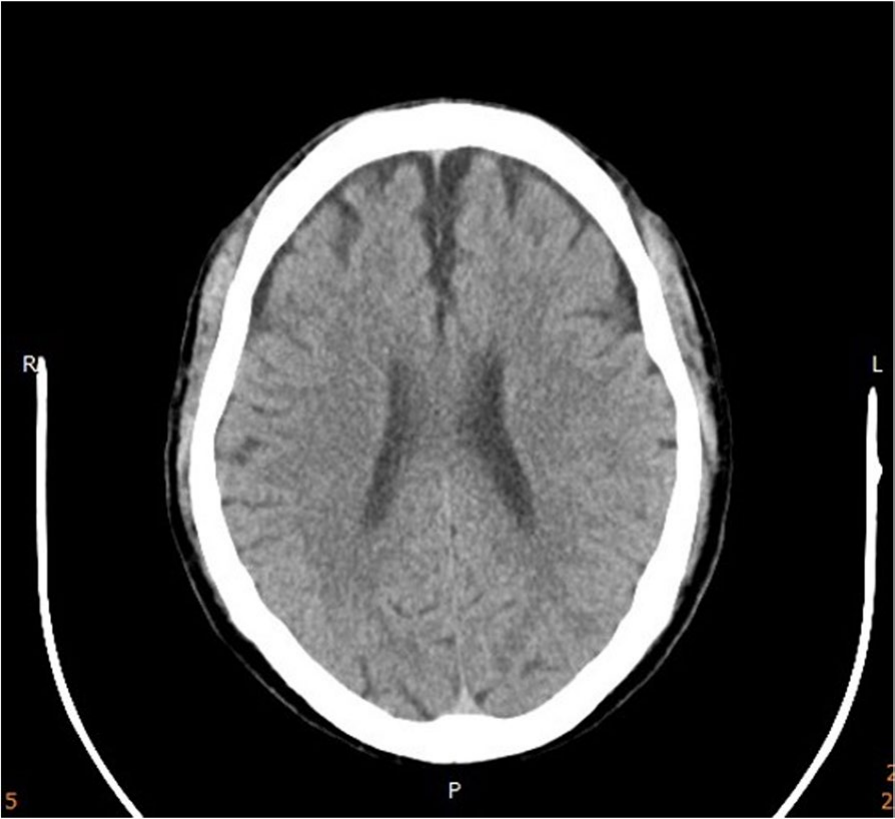
Post-seizure brain CT: Brain CT did not reveal remarkable bleeding or space occupying lesions

Upon further evaluation, brain computed tomography did not reveal any space-occupying lesion or bleeding (shown in Fig. 1). The serum biochemistry yielded the following results: sodium was 111 mEq/L, potassium was 3.0 mEq/L, total calcium was 7.6 mg/dl, albumin was 3.9 g/dl, phosphate was 2.1 mg/dL, blood urea nitrogen was 5.0 mg/dl, creatinine was 0.4 mg/dl, creatinine kinase was 1427 U/L, creatinine kinase-MB was 65 U/L, and troponin-T was 0.04 ng/ml. Hyponatremia, hypokalemia, hypophosphatemia, and hypocalcemia were indicated. The fasting blood glucose level was 174 mg/dl, thyroid-stimulating hormone was 1.51 ulU/mL, free thyroxine (free T4) was 1.21 ng/dl, cortisol at 4 PM was 25.17 μg/dL, and adrenocorticotropic hormone was 9.3 pg/ml, serum osmolality was 233 mOsm/L. A diagnosis of seizure secondary to acute severe hyponatremia was made. To correct his serum sodium, he was given intravenous injection of 3% saline. Urine osmolality measured a couple hours into treatment was low at 87 mOsm/L.

Patient received a total of 1 L of 3% saline over the first 24 hrs, and serum sodium increased from 111 mEq/L to 119 mEq/L. He was then started on normal saline for treatment. At 48 hrs, his serum sodium level was 126 mEq/L, with serum osmolality at 262 mOsm/L. Serum sodium was 133 mEq/L after three days of treatment. During this period, he received at total of 5 L IV fluid and made 11 L of urine. He was successfully extubated and there was no associated neurologic deficit. The patient’s only complaint was generalized muscle pain. According to the patient, he had been nervous about the colonoscopy and wanted to ensure adequate bowel preparation by drinking a large amount of water in addition to his bowel preparation regimen. He reported a total water consumption of 5.5 L on the morning of the scheduled examination. With his medication history, excessive water intake, and low urine osmolality, the patient’s acute hyponatremia was believed to be due to water intoxication facilitated by the use of desmopressin.

## Discussion and conclusions

Adequate bowel preparation prior to colonoscopy not only is critical for the quality of the examination but also ensures patient safety. Increased risks of adverse events, failed detection of malignant lesions, as well as need for repeated colonoscopy are directly linked to poor bowel cleansing. Several bowel preparation regimens have been developed with the ultimate goal of being effective, safe, and tolerable for patients.

Currently, the most commonly used bowel preparation agents are oral PEG and magnesium citrate with sodium picosulfate (MCSP). PEG is a water-soluble, non-absorbable substance that can pass through the gastrointestinal tract without movement of fluid and electrolytes [7]. The origin 4 l PEG solution regimen has been used throughout the years while other low-volume as well as split dose regimens were developed to improve patient acceptability. Alternatively, MCSP is a combination of stimulant and osmotic laxatives that has gain popularity due to better patient tolerance compared to PEG [1]. Both PEG and MCSP regimens have been proven to be safe and effective, and are recommended by current guidelines for bowel preparation prior to colonoscopic examinations [1, 2].

Although generally considered safe, all regimens are associated with risk of dehydration and potential electrolyte imbalances [1]. The most commonly discussed electrolyte imbalance is hyponatremia. Symptoms of hyponatremia can range from headache, nausea, vomiting to seizures, coma, and respiratory arrest. Old age and the presence of underlying diseases such as hypothyroidism, adrenal insufficiency, and kidney disease are known to increase risk of hyponatremia during bowel preparation. However, other mechanisms have been described in previous literature. Hypovolemic hyponatremia due to dehydration from severe vomiting and diarrhea in elderly patients using thiazide diuretics have been r repeatedly reported [4, 8]. Another established cause of severe hyponatremia is increased secretion of ADH [9]. With inappropriately high ADH, water excretion from the kidneys is impaired, resulting in water retention leading to hyponatremia. Increased release of ADH linked to non-osmolar stimuli such as nausea, pain, and anxiety were found in patients after bowel preparation [10]. Despite this finding, most patients with increased ADH had normal levels of serum sodium [9]. Severe hyponatremia was also reported in patients taking selective serotonin reuptake inhibitors (SSRIs), which was believed to be associated with syndrome of inappropriate antidiuretic hormone (SIADH) [11, 12]. In addition to the use of medications, excessive water ingestion without appropriate electrolyte intake for bowel preparation has also been concluded in causing non-osmotic stimuli, leading to inappropriately elevated ADH levels [9, 11, 13].

Our case described a healthy adult patient without serious comorbidities whose only significant history was benign prostate hyperplasia under desmopressin treatment for urinary frequency and nocturia. Desmopressin is a synthetic analogue of ADH often used to treat diabetes insipidus, primary nocturnal anuresis, and nocturia. Severe acute hyponatremia developed in our patient after ingestion of large volumes of water, subsequently resulting in seizure. The use of desmopressin likely contributed to fluid retention, making the patient vulnerable to water intoxication after ingestion of large amounts of water in a short period of time. Achinger et al. described a similar case of hyponatremia secondary to increased fluid intake in the setting of desmopressin use. Urine osmolality was over 500 mOsm/L at presentation but decreased to less than 100 mOsm/L after several hours of 3% saline treatment [14]. Urine osmolality after treatment will likely not be representative of ADH activity, explaining our patient’s low urine osmolality hours post-treatment. Although serum ADH level was not measured, elevated ADH from non-osmotic stimuli can be speculated to also play a role in the development of his hyponatremia.

To our knowledge, this is the first reported case of water intoxication during bowel preparation facilitated by use of desmopressin. Water intoxication in patients using desmopressin has been described in literature [15], however, never in the setting of bowel preparation. Patients are often instructed to drink enough water to prevent dehydration and electrolyte disturbances when taking bowel preparation agents. Nonetheless, excessive water intake in vulnerable patients can lead to hyponatremia due to water intoxication. Therefore, as highlighted in current guidelines for bowel preparation, regimens should be individualized [1, 2]. Proper patient education is also crucial in preventing undesired complications. Clinical awareness as well as prompt measurement of serum electrolytes should also be implemented in patients exhibiting symptoms concerning of imbalances. With a high suspicion of acute hyponatremia, colonoscopy should not be initiated and confirmation as well as treatment must not be delayed.

During history taking, in addition to information such as the use of diuretics, SSRIs, and pre-existing comorbidities that might contribute to electrolyte imbalance, the use of desmopressin should be considered a risk factor for development of hyponatremia secondary to water-intoxication during bowel preparation. For individuals susceptible to water-intoxication, bowel preparation solutions should contain adequate electrolytes and excessive fluid intake should be avoided.

In conclusion, acute hyponatremia due to bowel preparation is a rare yet serious complication that should not be neglected. Elevated serum ADH levels often play a critical role in the setting of water intoxication [11, 13]. The use of desmopressin, a synthetic analogue of ADH, can precipitate the development of water intoxication with hyponatremia. Thorough and meticulous history taking of comorbidities and current medications is necessary to guide clinicians in choosing the most appropriate bowel preparation regimen for each patient. Ensuring sufficient intake of electrolytes during bowel preparation and proper patient education are especially crucial for individuals that are vulnerable to water intoxication.

## Data Availability

The datasets used and/or analysed during the current study are available from the corresponding author on reasonable request.

## Abbreviations

PEG: Polyethylene glycol
ADH: Antidiuretic hormone
MCSP: Magnesium citrate with sodium picosulfate
SSRI: Selective serotonin reuptake inhibitor
SIADH: Syndrome of inappropriate antidiuretic hormone
